# Supplementary material for: Family Caregiving during the COVID-19 Pandemic in Canada: A Mediation Analysis
Source: Int J Environ Res Public Health. 2022 Jul 15;19(14):8636. doi: 10.3390/ijerph19148636 (PMC9317413; doi:10.3390/ijerph19148636)
Supplement: Supplementary file 1 [file ijerph-19-08636-s001.zip › Supplementary File S2--S6.pdf]

International Journal of
Environmental Research and Public Health

MDPI

Supplementary File S2: Self-Report version of the Clinical Frailty Scale

1. I am active, energetic, and exercise regularly.
2. I am well, but only occasionally active. I can manage finances, transportation, and heavy housework on my own.
3. My health conditions are well managed, but I am generally inactive. I may require advice on how to obtain supports with finances, transportation, or heavy housework
4. I am more tired than I used to be, and have more trouble obtaining supports than before, but can still coordinate things myself.
5. I need physical or practical assistance with finances, transportation, or heavy housework.
6. I need assistance with out of home activities, require help with bathing or medications, or struggle with stairs.
7. I need help with all of my personal care.
8. I am completely dependent for all of my personal care.
9. I am terminally ill and at the end of my life

Supplementary File S3: Figure S1 Scatterplot with Loess curve to check homoscedasticity assumptions

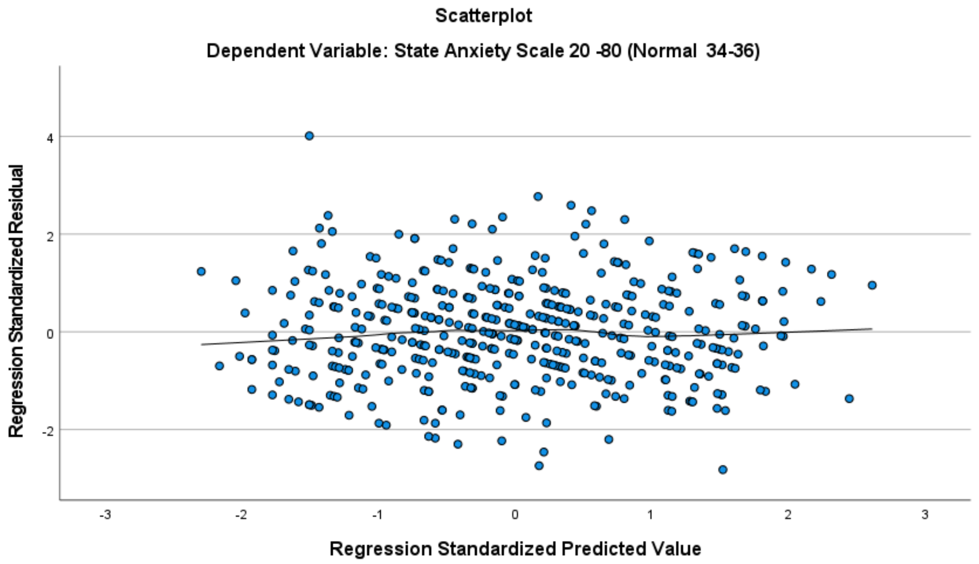

Supplementary File S4: Figure S2 P-P Plot, checking for normality of estimation error assumption

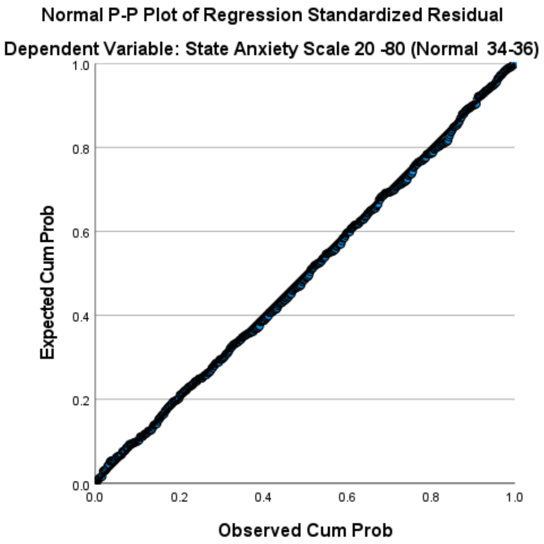

Supplementary File S5: Table S1 Correlations among key variables.

	1	2	3	4	5	6	7
1. State Anxiety Scale	1.000						
2. Age	-.206***	1.000					
3. Gender	-.035	-.116	1.000				
4. Weekly care time	.309***	-.019	-.003	1.000			
5. Navigation	.223***	-.043	.178***	.097*	1.000		
6. Financial difficulty	.418***	-.265***	.017	.344***	.155***	1.000	
7. Frailty	.422***	-.127**	.068	.271***	.233***	.341***	1.000
8. Social loneliness	.393***	-.062	.048	.182***	.125**	.232***	.312 ***

*p < .05 **p <.005 ***p <.0005

Supplementary File S6: CHERRIES Checklist for Reporting Results of Internet E-Surveys

Design	Describe survey design	Section 2 Paragraph 1
IRB (Institutional Review Board) approval and informed consent process.	IRB approval.	Section 2.3.2 University Health Ethics Research Board.
	Informed consent.	Section 2.3.2 Implied Consent
	Data protection.	Section 2.3.2 REDCap secure data collection platform.
Development and pre-testing.	Development and testing.	Section 2.4 Questionnaire reviewed by the research team and then Online REDCap survey reviewed by 5 family caregivers.
	Open survey versus closed survey.	Section 2.4 open survey
	Contact mode.	Section 2 .3.2
Recruitment process and description of the sample having access to the questionnaire.	Advertising the survey.	Section 2.3.2
	Web/Email.	Participants directed to REDCap link.
Survey administration.	Context.	Delivered on REDCAP
	Mandatory/voluntary.	As per ethics, only first 2 qualifying questions were mandatory.
	Incentives.	Draw for 2, $50 gift cards, Explanation included in Ethics Information about the Survey (Supplementary File 1).
	Time/Date.	Section 2.3.2 June 21 to August 31, 2020.

	Randomization of items.	No.
	Adaptive questioning.	Yes, Branching questions in REDCap. e.g. questions about homecare or care location only to those answering yes to those situations.
	Number of Items.	Sections were displayed as one page in REDCap.
	Number of screens (pages).	8 sections/ 8 pages.
	Completeness check.	Yes, Completed by REDCap platform and then manually
	Review step.	Yes participants could review/change answers and REDCap download PDF of responses was enabled.
Response rates.	Unique site visitor.	As per ethics anonymity we did not gather IP addresses or use cookies.
	View rate (Ratio of unique survey visitors/unique site visitors).	Not possible to calculate.
	Participation rate (Ratio of unique visitors who agreed to participate/unique first survey page visitors),	Section 3 Results 1st paragraph 58.9% (highlighted),
	Completion rate (Ratio of users who finished the survey/users who agreed to participate),	Section 3.2.1 81.2%
	Cookies used,	As per ethics anonymity we did not use cookies,
Preventing multiple entries from the same individual,	IP check,	Not possible with this platform
	Log file analysis,	Checked for identical responses.

	Registration,	No,
Analysis,	Handling of incomplete questionnaires,	Those with less than 80% of the questions completed were excluded,
	Questionnaires submitted with an atypical timestamp	None identified,
	Statistical correction	No.
